# Computational Fluid Dynamics Analysis of the Effect of Plaques in the Left Coronary Artery

**DOI:** 10.1155/2012/504367

**Published:** 2012-02-12

**Authors:** Thanapong Chaichana, Zhonghua Sun, James Jewkes

**Affiliations:** ^1^Discipline of Medical Imaging, Department of Imaging and Applied Physics, Curtin University, GPO Box, U1987, Perth, WA 6845, Australia; ^2^Fluid Dynamics Research Group, Department of Mechanical Engineering, Curtin University, Perth, WA 6845, Australia

## Abstract

This study was to investigate the hemodynamic effect of simulated plaques in left coronary artery models, which were generated from a sample patient's data. Plaques were simulated and placed at the left main stem and the left anterior descending (LAD) to produce at least 60% coronary stenosis. Computational fluid dynamics analysis was performed to simulate realistic physiological conditions that reflect the *in vivo* cardiac hemodynamics, and comparison of wall shear stress (WSS) between Newtonian and non-Newtonian fluid models was performed. The pressure gradient (PSG) and flow velocities in the left coronary artery were measured and compared in the left coronary models with and without presence of plaques during cardiac cycle. Our results showed that the highest PSG was observed in stenotic regions caused by the plaques. Low flow velocity areas were found at postplaque locations in the left circumflex, LAD, and bifurcation. WSS at the stenotic locations was similar between the non-Newtonian and Newtonian models although some more details were observed with non-Newtonian model. There is a direct correlation between coronary plaques and subsequent hemodynamic changes, based on the simulation of plaques in the realistic coronary models.

## 1. Introduction

Coronary artery disease (CAD) is the leading cause of death in advanced countries. The most common cause of CAD is atherosclerosis which is caused by the presence of plaques on the artery wall, resulting in the lumen stenosis. Plaques have been particularly associated with blood clots and compromise blood flow to the myocardium. This occurs when the coronary plaques suddenly rupture; if a clot cannot be treated in time, then the heart muscle will be impaired due to ischemic changes, leading to myocardial ischemia or infarction or, more severely, necrosis [1]. Therefore, an early detection and diagnosis of CAD is particularly important for reduction of the mortality and subsequent complications [1].

The natural history of coronary plaque is dependent not only on the formation and progression of atherosclerosis, but also on the vascular remodelling response. If the local wall shear stress is low, a proliferative plaque will form. Local inflammatory response will stimulate the formation of so-called “vulnerable plaque” which is prone to rupture with superimposed thrombus formation. The vast majority of these inflamed high-risk plaques cannot be detected by anatomic and myocardial perfusion imaging. Since the progression and development of vulnerable plaque is associated with low wall shear stress and the presence of expansive remodelling, measurement of these characteristics *in vivo* will enable risk stratification for the entire coronary circulation [2].

The wall shear stress (WSS), wall pressure, and blood flow changes in the human body cannot be measured directly on blood vessels, whereas computational fluid dynamics (CFD) can provide alternative ways to diagnose CAD [3]. The WSS factor in the coronary artery is known to play a significant role in the early formation of CAD [4]. In addition, the WSS at the local vessel wall can demonstrate a predisposition for atherosclerosis development for various anatomical sections, thus enabling the prediction of coronary disease [5].

CFD allows for efficient and accurate computations of hemodynamic features of both normal and abnormal situations in the cardiovascular system, *in vivo* simulation of coronary artery flow changes [3–6]. CFD is different from medical imaging visualisation as medical imaging techniques such as coronary angiography or computed tomography angiography provide anatomic alterations of the coronary artery wall due to the presence of plaques, thus allowing only assessment of the degree of lumen changes such as stenosis or occlusion [7, 8]. In contrast, CFD analysis enables the identification of hemodynamic changes in the coronary artery, even before the plaques are actually formed at the artery wall or can occlude the vessels. Therefore, to some extent, CFD allows early detection of coronary artery disease and improves the understanding of the progression of plaques, which are considered of paramount importance to clinical treatment. The purpose of this study was to investigate the hemodynamic effect of plaques in the left coronary artery by using CFD analysis. Simulated plaques were inserted into the left main stem and left anterior descending coronary arteries (taken from a selected patient's data), and hemodynamic analysis was performed to correlate the effect of presence of plaques with subsequent flow changes to the coronary main and side branches.

## 2. Materials and Methods

### 2.1. Patient Data Selection for Generation of Left Coronary Artery Model

**Figure 1:**
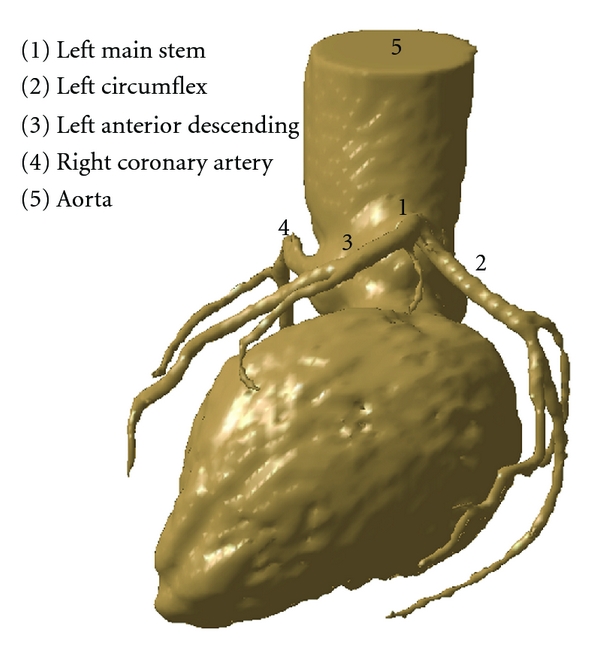
3D CT visualisation of a normal left coronary artery with side branches in a patient with suspected coronary artery disease.

A sample patient suspected of CAD who underwent multislice CT angiography was selected, and the patient's volume CT data was used to generate a 3D coronary model. The original CT data was saved in digital imaging and communication in medicine (DICOM) format and then transferred to a workstation equipped with Analyze 7.0 (Analyze Direct, Inc., Lexana, KS, USA) for image after-processing and segmentation. Three-dimensional (3D) volume data was postprocessed and segmented using a semiautomatic method with a CT number thresholding technique [9, 10], and manual editing was performed in some slices to remove soft tissues and artefacts. The segmented model was produced with a special focus on the left coronary artery (LCA) and its branches. The 3D LCA model was saved in “STL format” for further reconstruction purposes.  Figure 1 shows the anatomical details of the left coronary artery.

### 2.2. Realistic Plaques Modelling

**Figure 2:**
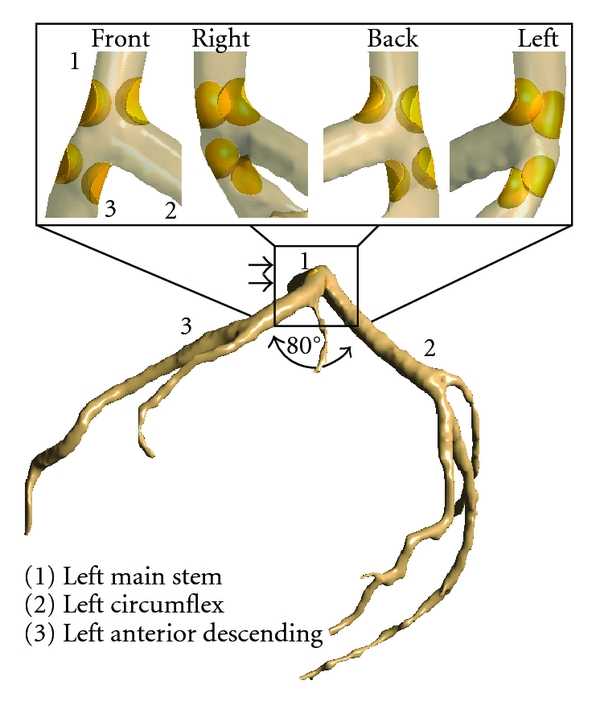
Plaque distribution in left coronary artery model is simulated at the left main stem and ostium of left anterior descending. Double arrows indicate that rectangle is an effective plaque location (EPL).

The actual plaques and degree of lumen stenosis on coronary artery wall were simulated at the left main stem (LMS) and the left anterior descending (LAD), as these artery branches are the common locations where plaques tend to form and induce myocardial ischemic changes [7, 11]. The plaques produced a lumen narrowing of approximately 60% diameter at the LMS and LAD, since more than 50% lumen stenosis leads to significant hemodynamic changes to flow within the coronary artery [12].  Figure 2 is the segmented LCA model showing various views of the position of the plaques at the left coronary artery.

### 2.3. Generation of Computational Models

The surface of LCA model with and without plaques (Figure 2) was prepared by using Blender version 2.48 (Blender Institute, Amsterdam, Netherlands). A gentle B-spline smoothing technique was applied between the left main trunk and side branches to reduce any potential nonphysical behaviour induced by sharp edges [13]. The surface models consisting of plaques and normal coronary arteries were converted into solid models and saved in “STL format” for the additional creation of meshing elements. Both models were used to create hexahedral and tetrahedral meshes to perform the CFD simulations. The hexahedral mesh configuration for the LCA model without plaques was 949,289 elements and 1,062,280 nodes, while the hexahedral mesh configuration for the LCA model with plaques was 928,311 elements and 1,041,936 nodes. The tetrahedral mesh configuration was 15,519 nodes and 78,618 elements. The meshes were generated using ANSYS ICEM CFD version 12 (ANSYS, Inc., Canonsburg, PA, USA), with details having been described in previous studies [6, 14, 15]. Finally, both mesh models were saved in “GTM format” for CFD computation.

### 2.4. Application of Physiological Parameters

**Figure 3:**
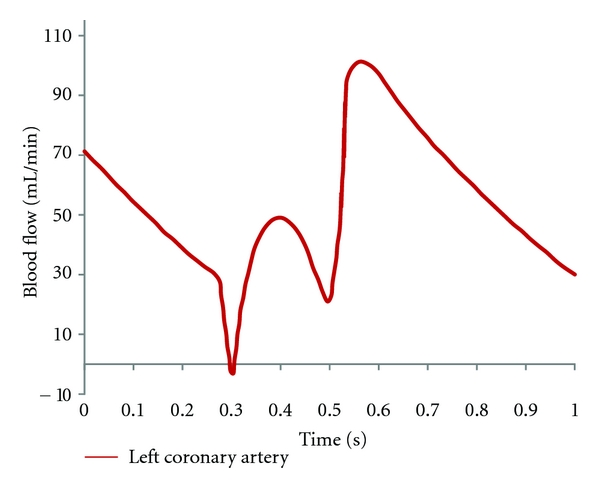
Cardiac pulsatile velocity at left main stem is applied for computational fluid dynamic simulation at the left coronary artery.

In order to ensure that our analysis reflects the realistic simulation of *in vivo* conditions, realistic physiological boundary conditions were applied for 3D numerical analysis. The transient simulation was performed using accurate hemodynamic rheological and material properties, as described in a previous study [16].  Figure 3 shows the pulsatile flow rates [17] at the aorta, reconstructed using a Fourier series [18] in Matlab (MathWorks, Inc. Natick, MA, USA). This Fourier series was applied using ANSYS CFX Command Language programming to define velocity and pressure boundary conditions. Pulsatile velocity was applied as an inlet boundary condition at the left main stem, and a zero pressure gradient was applied at the left anterior descending and left circumflex outlet boundaries [19]. Appropriate rheological parameters were applied with a blood density of 1060 kg/m^3^ and blood viscosity of 0.0035 Pa s [20, 21]. The blood flow was assumed to be laminar and a no-slip condition was applied at the walls. Plaque was assumed to be a rigid body [22]. Blood was assumed to be a Newtonian and incompressible fluid [4, 23]. In addition, the comparison of WSS between Newtonian and non-Newtonian models has been considered, especially at the stenotic locations [24]. A non-Newtonian blood model was simulated using the generalized power law [4, 25] which is defined as

(1) $$\begin{array}{c} \mu =\lambda \left(\dot{\gamma }\right){\left|\dot{\gamma }\right|}^{n(\dot{\gamma })-1},\\ \lambda \left(\dot{\gamma }\right)=\mu_{\infty }+\Delta \mu \;\exp\left[-\left(1+\frac{\left|\dot{\gamma }\right|}{a}\right)\exp\left(\frac{-b}{\left|\dot{\gamma }\right|}\right)\right],\\ n\left(\dot{\gamma }\right)=n_{\infty }-\Delta n\;\exp\left[-\left(1+\frac{\left|\dot{\gamma }\right|}{c}\right)\exp\left(\frac{-d}{\left|\dot{\gamma }\right|}\right)\right], \end{array}$$

where *μ*_*∞*_ = 0.035, *n*_*∞*_ = 1.0, Δ*μ* = 0.25, Δ*n* = 0.45, *a* = 50, *b* = 3, *c* = 50, and *d* = 4. Generalized power law model fits experimental stress-strain measurements over the range of strain rates, $\dot{\gamma },\;0.1<\;\dot{\gamma }<1000$ s^−1^ [25].

### 2.5. Performance of Computational Hemodynamic Analysis

**Figure 4:**
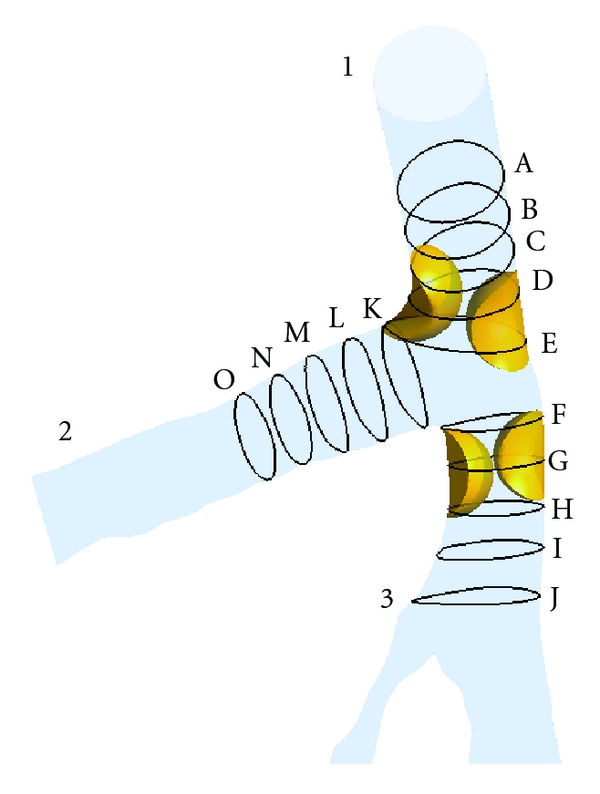
The EPL posterior view at left coronary artery model represents the cross-sectional positions of pre- and postplaque simulated models.

The Navier-Stokes equations were solved using the ANSYS CFX CFD package (version 12—ANSYS, Inc.), on a Microsoft Windows 7 32-bit machine, 6 MB RAM with an Xeon W3505 2.53 GHz CPU. The CFD simulation was run for 80 timesteps, representing 1.0 second of pulsatile flow, (0.0125 seconds per timestep), with each timestep converged to a residual target of less than 1 × 10^−4^ by approximately 100 iterations. The CFD solution was fully converged by approximately 8,000 time iterations per LCA model. The calculation time for each LCA model was approximately 2 hours. The configuration of this simulation is similar to previously published simulations [6, 14, 15]. Flow velocity, cross-sections of velocity pattern, and pressure gradient were calculated and visualised using ANSYS CFD-Post version 12 (ANSYS, Inc.).  Figure 4 represents the area of interest at the left coronary bifurcation and shows measurement positions of cross-sections of the models with and without plaques. The sectional planes were separated into 3 groups: Sections A–E, Sections F–J, and Sections K–O. The distance between sections in each group was approximately 0.5 millimetres. The parameter used to characterise the impact of plaques at the coronary bifurcation on hemodynamic flow was calculated as the magnitude of local pressure gradient [26, 27], which is defined as

(2) $$\text{PSG}=\sqrt{{\left(\frac{\partial p}{\partial_{u}}\right)}^{2}+{\left(\frac{\partial p}{\partial_{v}}\right)}^{2}+{\left(\frac{\partial p}{\partial_{w}}\right)}^{2}},$$

where *p* is the pressure in the area of interest, *u*, *v*, and *w* are the Cartesian *x*, *y*, and *z* coordinates in the direction of blood flow velocity. The local PSG is calculated by taking the time derivative of the local pressure. Finally, the value of PSG oscillated in relation to the percentage of plaques in the coronary lumen [28].

## 3. Results

The realistic left coronary artery models with plaques and without plaques were successfully performed with CFD analysis under *in vivo* physiological conditions during the systolic and diastolic phases. Peak systolic velocity and pressure were reached at a time of 0.4 sec, and middiastolic phase was reached at a time of 0.7 sec during the cardiac cycles, respectively. The analysis demonstrates a strong relationship between hemodynamic change and plaques at the left coronary artery.

### 3.1. CFD Analysis of the Left Coronary Artery: 2D Visualisation

**Figure 5:**
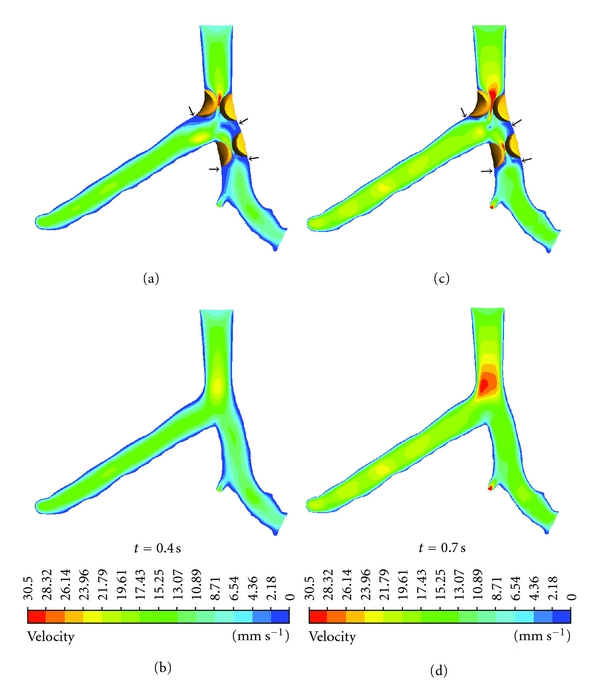
Flow velocity observed in pre- and postplaque simulated models during systolic peak of 0.4 s and middiastolic phase of 0.7 s. Arrows indicate the anatomic locations where plaques could spread into areas with low flow velocity.

**Figure 6:**
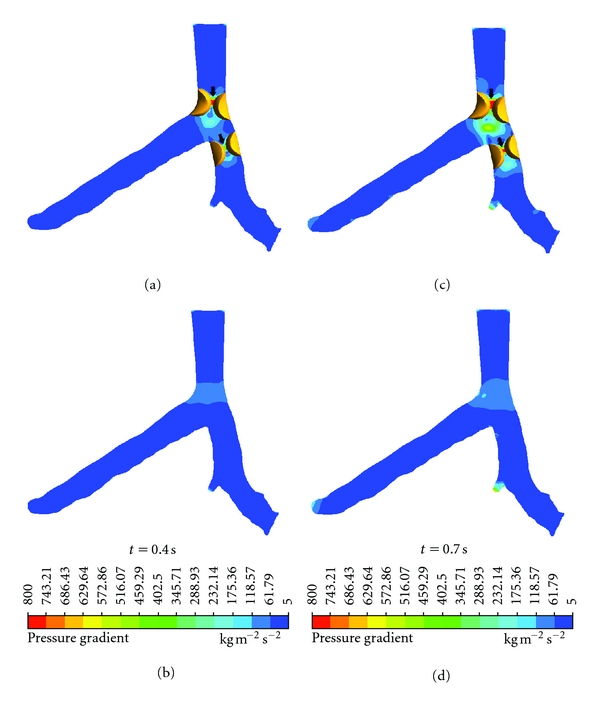
Pressure gradient observed in coronary models with and without plaques during systolic peak of 0.4 s and middiastolic phase of 0.7 s. Arrows indicate the high PSG locations where plaques may induce potential rupture or further atherosclerotic changes.

Flow velocity increased significantly in the presence of plaques due to resultant lumen stenosis. Poststenotic recirculation was observed in the LMS and LAD according to the CFD analysis, at the locations where plaques were present, as shown in Figure 5. Similarly, the pressure gradient (PSG) increased significantly at the LMS and LAD ostium, as shown in Figure 6. Measured PSG values at peak systolic and diastolic phases ranged from 459.29–800 kg/m^2^ s^2^ to 345.71–629.64 kg/m^2^ s^2^, corresponding to the LMS, LAD, and LCx in the presence of plaques. In contrast, in the absence of plaques, measured PSG values were significantly lower than those measured with presence of plaques, and these values ranged from 61.79–118.57 kg/m^2^ s^2^ to 5–61.79 kg/m^2^ s^2^.

### 3.2. CFD Analysis of Left Coronary Artery-Cutting Plane Visualisation

**Figure 7:**
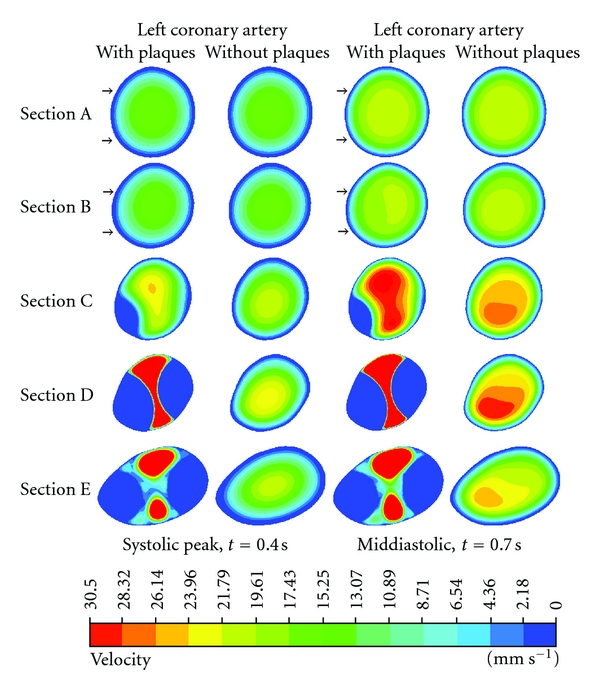
Cross-sectional views of A–E at the left main stem. Flow velocity observed with and without presence of plaque models during systolic peak of 0.4 s and middiastolic phase of 0.7 s. Arrows at Sections A and B refer to the normal flow pattern prior to the location of plaques, while flow pattern changes at the postplaque locations.

Flow velocity was visualised inside the LMS at Sections A–E, as shown in Figure 7. Flow patterns in both the pre and poststenotic cases were similar to those observed in Sections A and B (velocity ranged from 0 to 17.43 mm s^−1^). However, the flow velocity increased in Sections C–E (velocity ranged from 23.96 to 30.50 mm s^−1^), at the location of plaques during the systolic peak. In addition, the flow pattern was affected by the presence of plaques, which started from Sections A–E as observed in the poststenotic region, during the middiastolic phase, with velocity increasing from 28.32 to 30.50 mm s^−1^.

**Figure 8:**
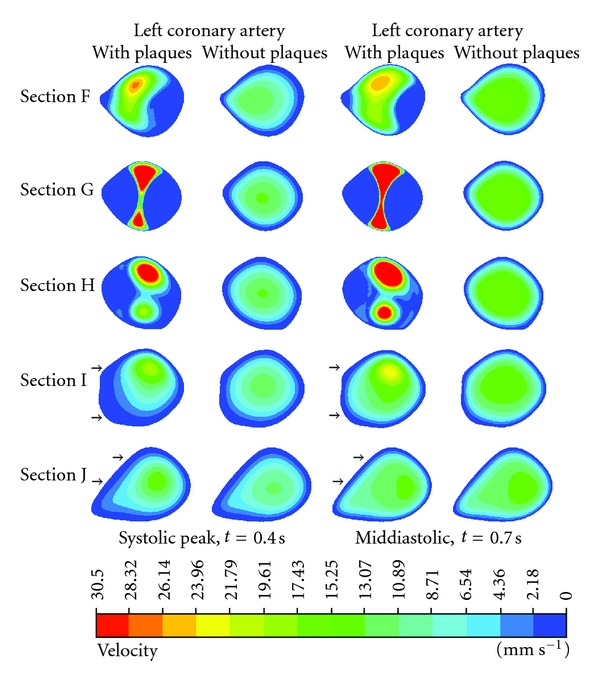
Cross-sectional views of F–J at the left anterior descending. Flow velocity observed with and without presence of plaque models during systolic peak of 0.4 s and middiastolic phase of 0.7 s. Arrows point to the low flow velocity areas at post-plaque locations due to interference of plaques.

Figure 8 demonstrates the hemodynamic effect of plaques inside the LAD with cutting views of Sections F–J. Poststenotic velocity reached its highest value in Sections F–H during peak systolic and middiastolic phases, with measured velocity ranging from 28.32 to 30.50 mm s^−1^. Furthermore, a recirculation region was apparent at the postplaque locations in Sections I and J. The velocity increased slightly with measured values ranging from 17.43 to 23.96 mm s^−1^ as observed in the post-stenotic regions.

**Figure 9:**
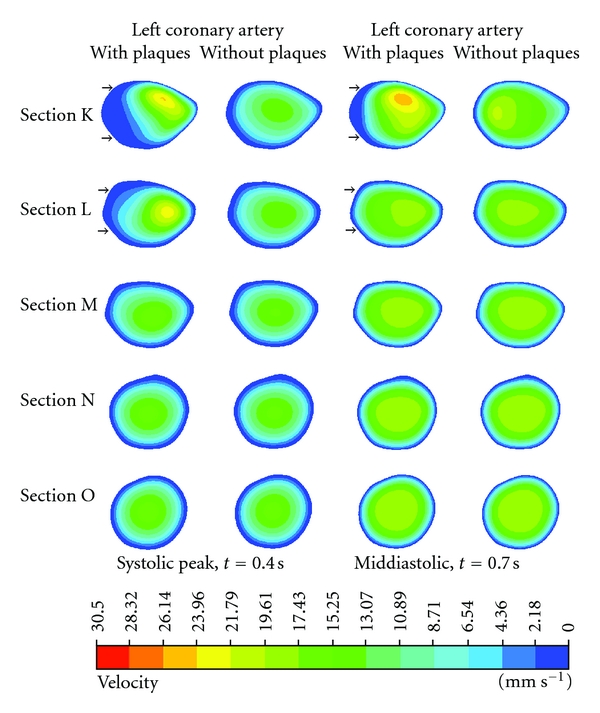
Cross-sectional views of K–O at the left circumflex. Flow velocity observed with and without presence of plaque models during systolic peak of 0.4 s and middiastolic phase of 0.7 s. Arrows indicate that the low flow velocity areas are present in the proximal segment of left circumflex due to the effect of plaques located at the left main stem.

Figure 9 represents the result of flow changes observed in the LCx from where plaques were situated in the LMS. Again, the recirculation location was obviously present in Sections K and L, located at postplaque positions. Flow velocity was found to slightly increase, ranging from 17.43 to 26.14 mm s^−1^ in both systolic peak and middle diastolic phases. Furthermore, velocity changes were not observed in Sections M–O as shown in the pre and post-stenotic regions with very similar flow patterns and velocity measured ranging from 0 to 21.79 mm s^−1^.

### 3.3. CFD Analysis of the Left Coronary Artery: Wall Shear Stress Comparisons

**Figure 10:**
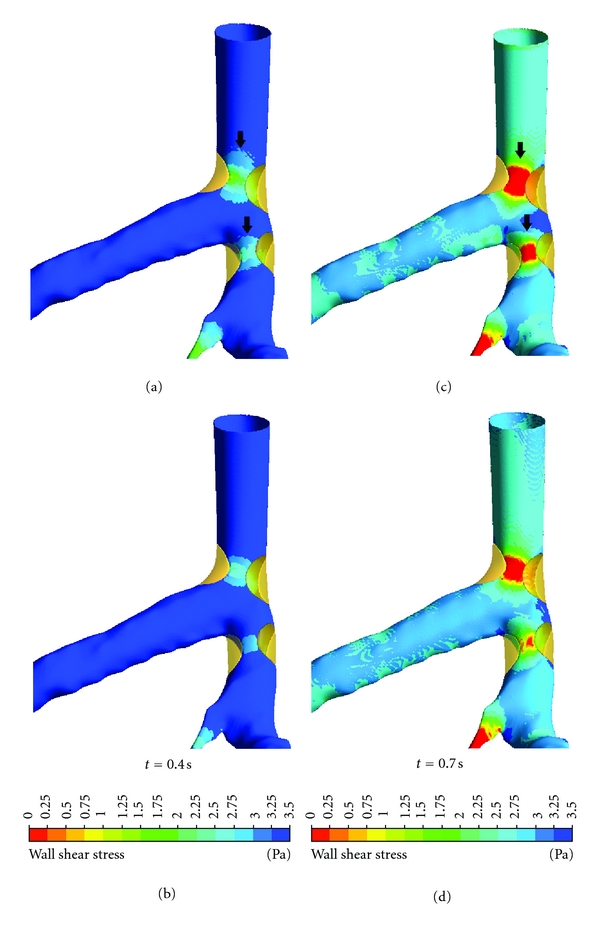
Comparison of WSS between non-Newtonian (a, c) and Newtonian (b, d) models observed in realistic coronary artery with presence of plaques during systolic peak of 0.4 s and middiastolic phase of 0.7 s. Arrows identify the different WSS at stenotic locations.

Analysis of WSS was particularly focused at the stenotic locations with comparison of non-Newtonian and Newtonian fluid models.  Figure 10 compares WSS with different fluid viscosities at the left coronary model with presence of plaques. WSS contour values ranged from 0 Pa to 3.50 Pa as observed in both fluid viscosity models. WSS was different due to presence of plaques at LMS branch at peak systolic phase, ranging from 0.50 Pa to 1.75 Pa with non-Newtonian model (Figure  10(a)) and ranging from 0.50 Pa to 1.0 Pa with Newtonian model (Figure  10(b)). Similar results of WSS values ranging from 1.50 Pa to 3.50 Pa with both viscosity models (Figures  10(c) and  10(d)) were found at middiastolic phase at plaques positions in LMS branch. WSS changes at stenotic locations in LAD were compared at peak systolic phases, ranging from 0.50 Pa to 1.0 Pa with non-Newtonian model (Figure  10(a)) and from 0.50 Pa to 0.75 Pa with Newtonian model (Figure  10(b)). WSS values at plaques positions in LAD were compared at middiastolic phases, ranging from 1.50 Pa to 3.50 Pa with non-Newtonian model (Figure  10(c)) and from 1.50 Pa to 3.25 Pa with Newtonian model (Figure  10(d)).

## 4. Discussion

This study shows that coronary plaques produce a significant impact on the subsequent flow changes in the coronary artery, in addition to the local hemodynamic interference due to the presence of plaques. This is clinically important as further potential effects could result from the plaques' interference, leading to adverse effects on the coronary artery, such as lumen stenosis or worsening of atherosclerosis.

It is well known that plaques most commonly form in the coronary bifurcation and coronary angulation, and that this is an important factor that has been found to be related to the development of atherosclerosis, as confirmed by our and other studies [6, 12, 29–31]. Multislice CT angiography and intravascular ultrasound have been widely used to detect and characterise plaques in the coronary arteries [7, 32]. Despite promising results having been achieved with imaging modalities, the limitations of these techniques were restricted to image visualisation and identification of coronary lumen changes due to presence of plaques, and no information is available about the interference of plaques with blood flow. In contrast, CFD overcomes those limitations by enabling the analysis of coronary blood flow and rheological factors [6, 14, 15]. This study investigated two important factors: PSG and flow velocity and qualified the impact of plaques on flow changes to the coronary arteries. The static wall pressure does not reflect the velocity profile from the flow axis to the blood wall [27, 33]. In the clinical situation, the PSG magnitude has been used to judge the risk of severity of plaques [28]. The highest PSG area may be relevant to potential coronary plaque rupture. In this study, the CFD analysis of the LCA with presence of plaques showed that the highest PSG was displayed in the locations at both LMS and LAD where plaques were simulated (Figure 6), with measured PSG value ranging from 743.21 to 800 kg/m^2^ s^2^.

The presence of plaques in the coronary artery is responsible for obstructing blood flow to the myocardium, consequently affecting the flow velocity [27]. Moreover, plaques influencing hemodynamic change may lead to the further distribution of plaques. Since velocity is the main component of local WSS and acts in the same direction as local WSS, which means that flow velocity is low when the WSS is low, as observed in a previous study [6], our analysis in this study has proposed explicitly hemodynamic changes inside the LCA surrounding the plaque locations (the so-called effective plaque location (EPL)) (Figure 2). In Sections A–E (Figure 7), we found that the flow velocity fluctuated in post-stenotic regions during cardiac cycles, and this could lead to abnormalities at the coronary wall, responsible for atherosclerosis. In Sections I–L (Figures 8 and 9), flow recirculation occurred, and the region of low velocity was observed within a short distance from the plaques. Consequently, plaques could generate an effect that spread into an area of low flow velocity as demonstrated in Sections I–L, matching with an area of low velocity in Figure 5, with measured low velocity value ranging from 0 to 2.18 mm s^−1^. This is confirmed by our previous analysis [6] showing that progression of plaques developed at a low-flow region. Our analysis provides insight into the effect of plaques on subsequent coronary flow changes although further studies are needed to verify our preliminary findings. WSS in non-Newtonian model was found to be similar to that observed in Newtonian model at plaques locations although more details were demonstrated in non-Newtonian model, as shown in Figure 10. The effect of plaques in left coronary is obviously shown in Newtonian model, and this is adequate for analysis of the plaque effect. The comparison of WSS between different viscosity models is confirmed by previous studies [4, 24]. A non-Newtonian model was simulated using the generalized power law as it has been reported to produce similar WSS effects to Newtonian model on coronary flow changes [4].

There are some limitations in our study that should be addressed. Firstly, realistic left coronary models, both pre and post-stenotic, were assumed to have a rigid wall rather than elastic wall; therefore, the simulation does not fully reflect the realistic physiological situation as the coronary wall moves during cardiac cycles. Secondly, the assumption of a Newtonian blood model becomes important especially in low flow and low wall shear stress regions. Nevertheless, a previous study has shown that the assumption of a Newtonian model is reasonable in this configuration [4]. Thirdly, the realistic plaques position may be affected by left coronary side branches that have not been evaluated in this study. Thus, future studies will use coronary models with a more realistic idealized geometry, extended to evaluate the effect of side branches.

In conclusion, we studied the effect of simulated plaques in the realistic left coronary artery on hemodynamic changes at the locations of plaques, as well as pre and post-stenotic regions inside the coronary artery. There is a direct effect of plaques in the left coronary artery on hemodynamic changes such as recirculation flow, low flow velocity regions, wall shear stress, and wall pressure gradient, indicating the potential for plaques to rupture, causing atherosclerosis. Further studies focusing on the realistic plaque's effect on coronary side branches should be performed to verify our results.
